# Monoamine neurotransmitters and fibroblast growth factor-2 in the brains of rats with post-stroke depression

**DOI:** 10.3892/etm.2014.1674

**Published:** 2014-04-08

**Authors:** XIAO-WEI JI, CHUN-LING WU, XING-CHEN WANG, JIE LIU, JIAN-ZHONG BI, DIAN-YUN WANG

**Affiliations:** 1 Department of Neurology, Second Hospital Affiliated to Shandong University of Traditional Chinese Medicine, Jinan, Shandong 250001, P.R. China; 2 Blood Purification Center, Jinan Central Hospital Affiliated to Shandong University, Jinan, Shandong 250013, P.R. China; 3 Department of Neurology, The Second Hospital of Shandong University, Jinan, Shandong 250033, P.R. China; 4 Department of Traditional Chinese Medicine, Dongying People’s Hospital, Dongying, Shandong 257091, P.R. China

**Keywords:** post-stroke depression rat, serotonin, dopamine, norepinepherine, fibroblast growth factor-2

## Abstract

The aim of the present study was to investigate the changes in the levels of serotonin (5-HT), dopamine (DA), norepinephrine (NE) and fibroblast growth factor-2 (FGF-2) in the brains of rats with post-stroke depression (PSD). A rat model of stroke was established by middle cerebral artery occlusion and the rats were randomly divided into two groups: Control and modification groups. The rats in the modification group had PSD, while the rats in the control group had experienced a stroke only. The PSD model was established by applying chronic mild stress to the individually housed rats. High-performance liquid chromatography was used to detect the levels of 5-HT, DA and NE, while western blotting was used to detect the FGF-2 protein expression levels in the frontal lobe and hippocampus. Quantitative polymerase chain reaction was also used to determine the mRNA expression levels of FGF-2 in the frontal lobes of the two groups. The levels of 5-HT, DA and NE in the frontal lobe and hippocampus of the rats in the PSD group were significantly lower than the levels observed in the rats in the stroke group (P<0.01). In addition, protein expression levels of FGF-2 in the frontal lobe of the rats in the PSD group were significantly lower when compared with the control group rats (P<0.01), however, the protein expression levels of FGF-2 in the hippocampus did not exhibit a statistically significant difference (P>0.05). The mRNA expression levels of FGF-2 in the frontal lobe of the rats in the modification group were significantly lower than the levels in the control group rats (P<0.01). Therefore, reduced levels of monoamine neurotransmitters and FGF-2 expression in the brains of rats with PSD are associated with the incidence of PSD.

## Introduction

Cerebrovascular disease is a common disease that endangers human life and health. Ischemic stroke accounts for ~80% of the cases of cerebrovascular disease. Post-stroke depression (PSD) is a common complication of strokes, with an incidence of 25–80%, due to different subjects, different assessing periods after stroke, different diagnostic criteria and psychological testing methods, and different experiences of the researchers (1). The pathogenesis of PSD is complex and a number of the underlying mechanisms remain unclear.

The brain neurotransmitters, norepinephrine (NE), serotonin (5-HT) and dopamine (DA), are biogenic amines that transmit information between nerve cells or neurons and effector cells, integrating the overall coordination of human body functions. If these neurotransmitters are defected, the normal function of the nervous system is affected, resulting in depression (2–4). In the pathogenesis of depression, the function and content of these three types of monoamine neurotransmitter may change and affect the regulation of emotions to a varying extent.

Fibroblast growth factor (FGF) plays an important role in the development of the neocortex and the survival and growth of adult neurons. The main role of FGF in the brain is the regulation of neuronal and glial cell proliferation, migration, differentiation and survival (5). Precursor cells of the neural plate have the capacity to differentiate into serotonergic and dopaminergic neurons (6–8), and are considered to be associated with neural plasticity. Abnormal expression of FGF may lead to depression (9). FGF-2, one of the original types of ligand in the FGF family, protects ischemic cerebral tissue by activating the anti-apoptotic protein (10–12). However, whether there is an association between PSD and brain monoamine neurotransmitters and FGF remains unknown.

In the present study, the levels of NE, 5-HT, DA and FGF-2 protein were analyzed in the brains of rats with PSD in order to investigate the causes of PSD. The mRNA expression levels of FGF-2 were also analyzed to identify the molecular mechanisms underlying the change in the levels of FGF-2 protein. The aim of the study was to provide a biological basis for the diagnosis and treatment of PSD.

## Materials and methods

### Animals

White male Sprague-Dawley rats (weight, 250–300 g) were obtained from the Experimental Animal Center at Shandong University of Traditional Chinese Medicine (Jinan, China). A total of 45 rats were successfully induced as stroke models with a score of 1–3 (13). Scoring was as follows: behavior was completely normal, 0 points; the rats were held by their tails, the contralateral forelimb of surgery rotation, adduction, 1 point; the rats were placed on the ground, squeeze the sides to check their resistance, resistance decreased in the contralateral forelimb of surgery, 2 points; the rats were placed on the ground to observe the walk, circling around the contralateral of surgery, 3 points; the rats were seriously injured and unable to do independent activities, 4 points. The stroke rats were randomly divided into PSD and stroke groups. There was no statistically significant difference in the scores of the rats between the two groups. The rats in the stroke group were fed normally. In the PSD group, the animals were treated simultaneously with unpredicted chronic mild stress (CMS). The experiments were approved by the Animal Experimentation Ethics Board of Shandong Univiersity, which also meet the ethical requirements of the China National Act on the Use of Experimental Animals. All surgeries were performed under general anesthesia, and all efforts were made to minimize suffering. The rats were placed in individual cages and maintained in a 12:12 h light/dark cycle at a controlled temperature (21±2°C) and humidity (65±5%). The rats had free access to food and tap water.

### Chemicals and equipment

ABI-7500 Real-Time detector (ABI, Carlsbad, CA, USA), AB-160 electronic analytical balance (Sartorius AG, Beijing, China), micropipette, rabbit anti-FGF-2, horseradish peroxidase-conjugated AffiniPure goat anti-rabbit IgG (H+L; Shanghai BlueGene Biotech CO, Shanghai, China), 4% chloral hydrate (The Second Hospital Affiliated to Shandong University of Traditional Chinese Medicine, Jinan, China), perchloric acid, polyvinylidene difluoride (PVDF), sodium acetate, citric acid, methanol, *et al*.

### Modeling method

A rat stroke model was established as described previously with minor modifications (14). At day 7 following surgery, an unpredicted CMS procedure was performed, as described previously, to establish the rat PSD model (15,16). The CMS protocol consisted of the sequential application of a variety of mild stressors: i) Fasting for 20 h; ii) water deprivation for 17 h; iii) cage tilt (45°) for 17 h; iv) constant illumination for 17 h; v) wet cage (100 g sawdust + 200 ml water) for 21 h; vi) forced swimming at 4°C for 5 min; vii) horizontal shaking for 5 min; viii) immobilization for 2 h; and ix) tail clamping for 1 min. These stressors were randomly scheduled over a one-week period and repeated throughout the subsequent three weeks of the experiment. For the stroke group, the animals were left undisturbed in their home cages, except during housekeeping procedures such as cage cleaning.

Following three weeks of CMS application, the rats were anesthetized, rapidly decapitated and the brain tissue was quickly placed in the ice plate, including the frontal lobe and hippo-campus. The brain tissue was stored at −70°C until use. Before total RNA was extracted, the tissue was homogenized.

### Determination of the monoamine levels

For the measurement of DA, 5-HT and NE levels, the frozen brain tissue was thawed at room temperature and then filtered through 0.45-μm membranes. A 2–5 μl aliquot of each sample was injected into a high performance liquid chromatography column and the levels were detected electrochemically. The results of the qualitative analysis were the retention times of the sample and standard, while the results of the quantitative analysis were the peak areas of the sample and standard. All the results were recorded by the N2005 chromatography data workstation.

### Determination of the protein expression levels of FGF-2

Proteins were extracted from the prefrontal cortex and hippocampus for western blotting using a mixture of protein extraction reagent and phosphates. The protein concentration was determined using Coomassie Brilliant Blue dye. The denatured proteins (50 μg) were separated by 15% sodium dodecyl sulfate-polyacrylamide gel electrophoresis and electrotransferred onto PVDF membranes. The membranes were incubated overnight at 4°C with primary monoclonal rabbit anti-FGF-2 antibodies. Subsequently, horseradish peroxidase-conjugated secondary antibodies (goat anti-rabbit) were diluted 1:1,000 in Tris-buffered saline with Tween 20 and were applied to the membranes. Chemiluminescent detection was performed using enhanced chemiluminescence. For visualization and densitometric analysis, a Chemi-Doc XRS+ imaging system and Image Lab software were used (Bio-Rad, Hercules, CA, USA).

### Determination of mRNA expression levels of FGF-2

Total RNA from the frontal cortex tissue of the rats was extracted using TRIzol reagent, according to the manufacturer’s instructions. The isolated RNA was reverse transcribed into cDNA. The sample DNA that contained the target sequence was incubated at 95°C for 5 min. Subsequently, the temperature was lowered to 55°C for 5 min to anneal the target to the complementary sequence. The temperature was then raised to 72°C for 1 min to allow *Taq* polymerase to attach at each priming site and extend a new DNA strand. These steps were repeated for 40 cycles.

Aliquots of the polymerase chain reaction (PCR) products (10 μl) were size-separated by electrophoresis on a 2% agarose gel. Quantitative PCR was performed using SYBR Green Master mix and ROX reference dye (Kapa SYBR Fast qPCR kit; Kapa Biosystems), according to the manufacturer’s instructions. Briefly, cDNA was obtained by reverse transcription of the RNA from the brains of the rats. SYBR Green signals were detected by a Mx3000P™ Multiplex Quantitative PCR machine and the transcript levels were quantified using the Ct value method, where the values were normalized against the internal control, GAPDH. PCR products were analyzed by gel electrophoresis on a 1.5% agarose gel, and the specificity of amplification was confirmed by the melting curves. The primers employed in the reverse transcription and quantitative PCR analyses are listed in Table I.

### Statistical analysis

All the results are expressed as the mean ± standard deviation and statistical analysis was performed using Statistical Product and Service Solutions software, version 16.0 for Windows (SPSS, Inc., Chicago, IL, USA). Inter-group comparisons were performed using the Student’s t-test, assuming normal distribution, where P<0.05 was considered to indicate a statistically significant difference.

## Results

### Physiological parameters of the PSD and stroke groups

Physiological parameters, including the mean arterial pressure, blood pH, arterial oxygen and carbon dioxide tensions, hematocrit and blood glucose levels, were recorded and controlled within the normal ranges (17). No statistically significant differences in these parameters were identified between the PSD and stroke groups.

### Monoamine transmitter levels in the brains of the rats

Compared with the control group, the reduction in the levels of NE, 5-HT and DA in the frontal lobe of the rats in the PSD group was statistically significant (P<0.01; Student’s t-test; Table II).

The effect of PSD on the brain was further evaluated in terms of the levels of NE, 5-HT and DA in the hippocampus. When compared with the control stroke group, the reduction in the levels of NE, 5-HT and DA in the hippocampus of the rats in the PSD group was statistically significant (P<0.01; Student’s t-test; Table III)

### Protein expression levels of FGF-2 in the frontal lobe and hippocampus of the rats

It is well established that the addition of FGF-2 is critical to the palingenesis of nerves (18–21). Thus, the reduction in monoamine transmitters levels observed in the brains of the rats may be due to changes in the levels of FGF-2 expression in the frontal lobe and hippocampus. To further test this hypothesis, western blotting was used to analyze the protein expression levels of FGF-2 in the frontal lobe and hippocampus tissues of the rats.

**Figure 1:**
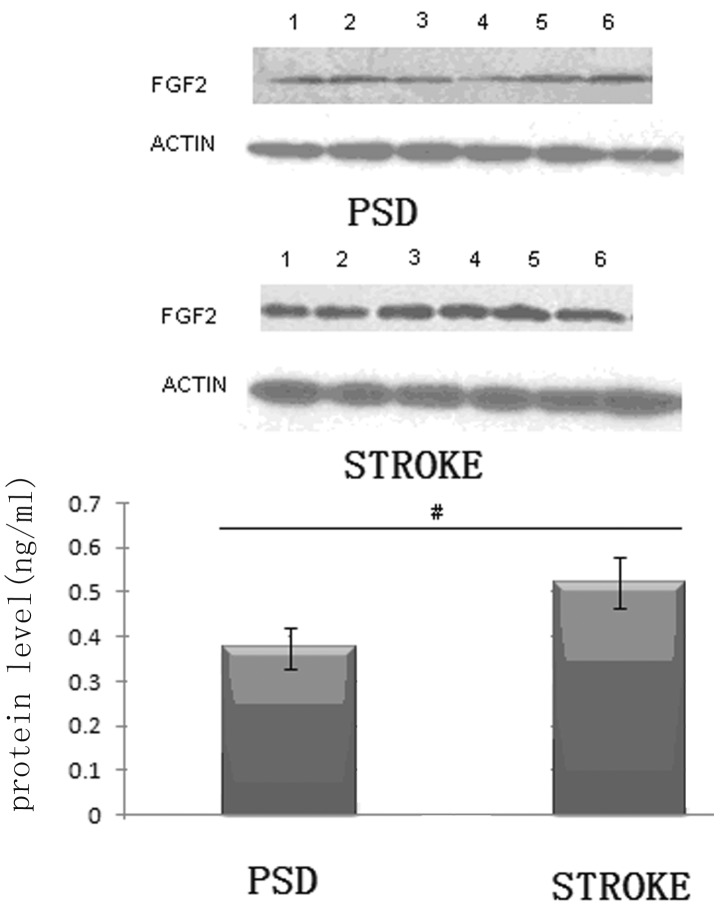
Protein expression levels of FGF-2 in the frontal lobe tissue. Lanes 1–6 represent individual rats in the same group. The bar graph shows the average number of PSD and STROKE in each condition. Results are presented as the mean ± standard deviation from six individual rats chosen at random. ^#^P<0.01, PSD vs. stroke. FGF-2, fibroblast growth factor-2; PSD, post-stroke depression; STROKE, control group.

**Figure 2:**
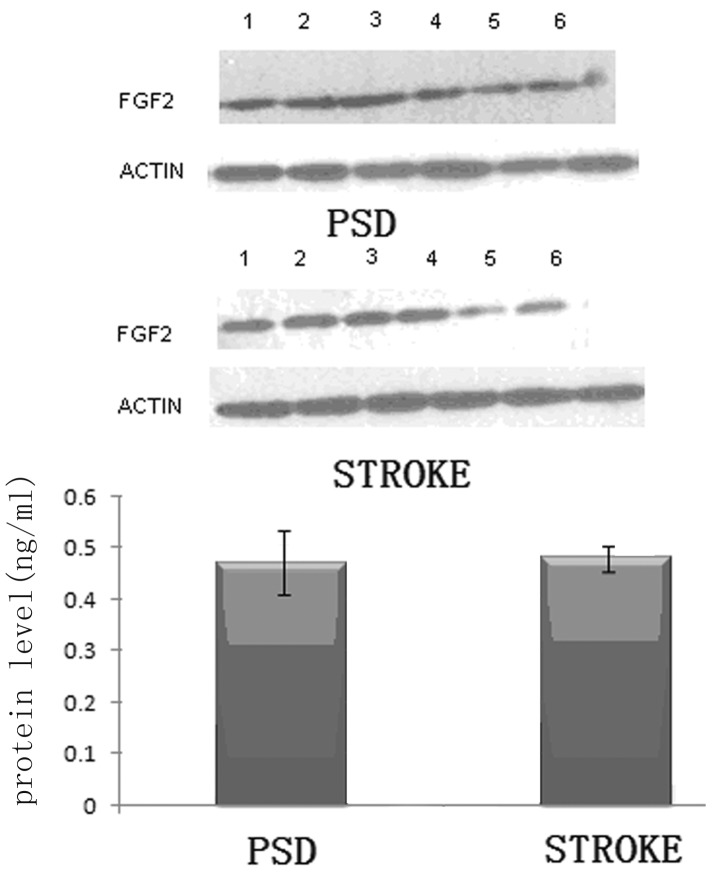
Protein expression levels of FGF-2 in the hippocampus. Lanes 1–6 represents a single individual in the same group. The bar graph shows the average number of PSD and STROKE in each condition. Results are presented as the mean ± standard deviation from six individual rats chosen at random. FGF-2, fibroblast growth factor-2; PSD, post-stroke depression; STROKE, control group.

In the frontal lobe tissue, the FGF-2 protein expression levels in the PSD group were significantly lower than those in the stroke group (P<0.01; Student’s t-test; Fig. 1). Subsequently, the same analysis was performed on the hippocampal tissue. Although the expression levels of FGF-2 appeared to be reduced in the hippocampus of the rats in the PSD group as compared with those in the stroke group, the decrease in the relative protein expression levels was not statistically significant (P>0.05; Student’s t-test; Fig. 2). The results demonstrate that in the frontal lobe, a correlation exists between the level of monoamine transmitters and FGF-2 protein expression. To further analyze this hypothesis, the mRNA expression levels of FGF-2 in the frontal lobe were determined.

### mRNA expression levels of FGF-2 in the frontal lobe of the rats

**Figure 3:**
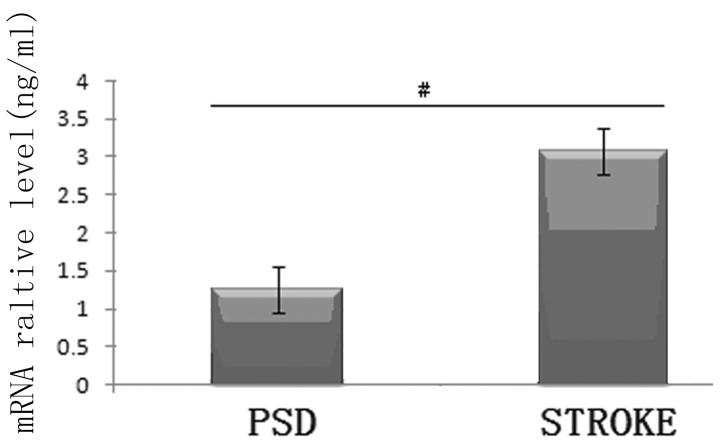
mRNA expression levels of FGF-2 in the frontal lobe tissue. ^#^P<0.01, PSD vs. stroke. FGF-2, fibroblast growth factor-2; PSD, post-stroke depression.

Compared with the stroke group, the mRNA expression levels of FGF-2 in the frontal cortex tissue of the PSD rats were significantly decreased (P<0.01; Student’s t-test; Fig. 3). This marked reduction in the frontal lobe of the rats indicated that the decreased FGF-2 protein expression levels, as observed in the previous experiment, were caused by the downregulation of FGF-2 mRNA expression.

## Discussion

As a secondary disease, PSD directly affects the quality of life and functional rehabilitation of patients, significantly reducing the desire for rehabilitation and delaying the functional recovery of nervous and cognitive aspects. PSD not only increases the mortality rate and rate of suicide, but also increases the burden on society and the family of the patient (22–29). The main theory underlying the pathogenesis of PSD is a series of abnormalities regarding neurotransmitters and endocrine and neurotropic factors following a stroke (30). These abnormalities cause a variety of psychological stresses. The interaction of physiological, psychological and social factors causes an imbalance of NE and 5-HT neurons in the feedback loop system formed by the frontotemporal lobe, basal ganglia, ventral brainstem and its pathway disruption. All these factors promote the occurrence of PSD (31,32). However, the cause of the reduction in the levels of monoamine transmitters may be complex and is yet to be completely understood.

In the present study, a stroke model was established in rats by adopting the method of occlusion of the left middle cerebral artery. The cerebrovascular pathological changes in the brains of the rats following this procedure are similar to clinical stroke pathology. Following the successful establishment of the rat stroke model, the rats underwent unpredictable CMS, which simulated the pathological state of patients with PSD. A significant reduction in the levels of 5-HT, NE and DA was observed in the frontal cortex and hippocampus of the rats with PSD, as compared with the rats in the control group. These results may be associated with the direct disruption of the pathways involving 5-HT and NE following a stroke. Neuronal cell bodies are located in the brainstem and the axons of these neuronal cell bodies reach the frontal cortex and hippocampus through the thalamus and basal ganglia, which play an important role in mood and emotion regulation. When these pathways involving 5-HT and NE are invaded by a lesion, reduced levels of 5-HT and NE lead to the occurrence of PSD.

It is well established that FGF-2 is necessary for progenitor cell proliferation in the developing brain *in vivo* (33–36), and FGF-2 stimulates the proliferation of neural stem cells and progenitors isolated from the embryonic brain (33,37–44). To further investigate the cellular mechanism underlying the significant reduction in the levels of monoamine transmitters in the frontal lobe and hippocampus of the rats, the protein expression levels of FGF-2 in the two parts of the brains were determined by western blot analysis. Through western blotting, the protein expression levels of FGF-2 in the frontal lobe were found to be significantly lower than those in the stroke group (P<0.01; Student’s t-test). By contrast, the reduction in the protein expression levels of FGF-2 in the hippocampus of the PSD group was not statistically significant when compared with the stroke group, although a slight decrease was observed (P>0.05; Student’s t-test). The results indicate that the FGF-2 signaling pathway may be the cause of the reduction in monoamine levels and the occurrence of PSD. Additional experiments, which detected the FGF-2 mRNA expression levels in the frontal lobe by quantitative PCR, revealed that the decreased protein expression levels of FGF-2 in the PSD group were caused by the decreased mRNA expression levels of FGF-2. The FGF signaling pathway consists of multiple ligands and receptors. The major role of FGF in the brain is the regulation of differentiation, proliferation, migration and survival of vascular endothelial cells, neurons and glial cells. The FGF signaling pathway also has the capacity to induce the differentiation of neural plate precursor cells into dopaminergic and serotonergic neurons, which plays an important role in brain development (11). A previous study (45) demonstrated that the dysfunction of the FGF system in patients with major depression is not induced by antidepressants. The results of the present study demonstrated that the decreased protein and mRNA expression levels of FGF-2 in the frontal lobe may be associated with the occurrence of PSD and the decreased levels of 5-HT, NE and DA. Accordingly, the observations of the present study may aid the understanding of the underlying mechanisms that lead to PSD under physiological and pathological conditions.

In conclusion, using a variety of assays, the present study has demonstrated that a reduction in FGF-2 mRNA expression levels is key to the reduced levels of monoamine neurotransmitters and the occurrence of PSD.

## Figures and Tables

**Table I:** Primer sequences and the length of PCR products.

Gene	Direction	Primer sequences	Amplified fragment length (bp)
FGF-2	Upstream	5′-TGCGCATCCATCCAGACGGC-3′	130
	Downstream	5′-GCCAGGTACCGGTTCGCACA-3′	
β-actin	Upstream	5′-AGAACATCATCCCTGCATCC-3′	112
	Downstream	5′-TGGATACATTGGGGGTAGGA-3′	

PCR, polymerase chain reaction; FGF-2, fibroblast growth factor-2.

**Table II:** Levels of NE, 5-HT and DA in the frontal lobe (n=8 per group; ng/ml).

Group	NE	5-HT	DA
PSD	173.9±13.2	349.9±21.9	233.9±12.1
Stroke	285.1±19.5	722.1±13.2	284.3±14.0
F-value	0.671	1.163	1.050
P-value	<0.01	<0.01	<0.01

NE, norepinephrine; 5-HT, serotonin; DA, dopamine; PSD, post-stroke depression.

**Table III:** Levels of NE, 5-HT and DA in the hippocampus (n=8 per group; ng/ml).

Group	NE	5-HT	DA
PSD	157.5±15.4	327.9±27.9	230.8±13.1
Stroke	271.2±18.4	725.9±15.4	285.4±15.6
F-value	0.962	2.175	0.485
P-value	<0.01	<0.01	<0.01

NE, norepinephrine; 5-HT, serotonin; DA, dopamine; PSD, post-stroke depression.
